# The Mechanistic Basis for Successful Spinal Cord Stimulation to Generate Steady Motor Outputs

**DOI:** 10.3389/fncel.2019.00359

**Published:** 2019-08-09

**Authors:** Amr A. Mahrous, Mohamed H. Mousa, Sherif M. Elbasiouny

**Affiliations:** ^1^Department of Neuroscience, Cell Biology, and Physiology, Boonshoft School of Medicine and College of Science and Mathematics, Wright State University, Dayton, OH, United States; ^2^Department of Physiology, Feinberg School of Medicine, Northwestern University, Chicago, IL, United States; ^3^Department of Biomedical, Industrial, and Human Factors Engineering, College of Engineering and Computer Science, Wright State University, Dayton, OH, United States

**Keywords:** spinal motoneurons, electrical stimulation, spinal cord injury, motor output, use-dependent adaptation, sensorimotor integration

## Abstract

Electrical stimulation of the spinal cord is a promising rehabilitation intervention to restore/augment motor function after spinal cord injury (SCI). Combining sensory feedback with stimulation of remaining motor circuits has been shown to be a prerequisite for the functional improvement of SCI patients. However, little is known about the cellular mechanisms potentially underlying this functional benefit in the injured spinal cord. Here, we combine computer simulations with an isolated whole-tissue adult mouse spinal cord preparation to examine synaptic, cellular, and system potentials measured from single motoneurons and ventral roots. The stimulation protocol included separate and combined activation of the sensory inputs (evoked by dorsal root stimulation) and motor inputs (evoked by stimulation of spinal cord tissue) at different frequencies, intensities, and neuromodulatory states. Our data show that, while sensory inputs exhibit short-term depression in response to a train of stimulation, motor inputs exhibit short-term facilitation. However, the concurrent activation of both inputs elicits a stronger and steadier motor output. This effect is enhanced by the application of pharmacological neuromodulators. Furthermore, sensorimotor excitatory postsynaptic potentials (EPSPs) summate sublinearly (i.e., their combination produces an excitatory potential smaller than the sum of the excitatory potentials they would individually produce). However, ventral root compound action potentials (CoAPs) summate supralinearly generating much higher outputs. Computer simulations revealed that the contrasting summation and disproportionality in plasticity between the excitatory postsynaptic potentials (EPSPs) and CoAPs result from the motoneuronal firing threshold acting as an amplitude-selective filter. Together, these results provide the mechanistic basis for the cellular processes contributing to the generation of steady motor outputs using spinal stimulation. This data has great potential to guide the design of more effective stimulation protocols in SCI patients.

## Introduction

Electrical stimulation of the spinal cord is currently showing promise for motor rehabilitation after spinal cord injury (SCI). With electrical stimulation, patients showed improved trunk control, standing, stepping, and urogenital function (Carhart et al., 2004; Harkema et al., 2011; Gerasimenko et al., 2015; Angeli et al., 2018). Notably, patients with complete SCI were able to stand and step with minimal assistance only when trains of electrical stimulation activating intrinsic motor pathways and circuits in the transected spinal cord was provided (Harkema et al., 2011; Gerasimenko et al., 2015; Angeli et al., 2018; Wagner et al., 2018). In these studies, SCI patients could not step or stand without electrical stimulation – even with manual facilitation or after several training sessions. This indicates the necessity of the electrically evoked motor potentials to the generation of these functional benefits. Importantly, electrical stimulation was not effective unless it included proprioceptive feedback generated from muscle length changes and load-bearing during stepping and standing, indicating that sensorimotor integration mediates these effects.

To investigate the effects of electrical stimulation after SCI at the synaptic, cellular, and system levels, we used electrophysiological recordings and computer simulations to study the plasticity, integration, and neuromodulation of electrically evoked sensory and motor synaptic potentials in an isolated spinal cord preparation. Specifically, our goals were to: (1) Identify the characteristics of electrically triggered sensory and motor synaptic potentials generated in motoneurons (synaptic level), (2) understand how electrically triggered sensory and motor synaptic potentials integrate within the motoneuron (cellular level), and (3) examine the transformation of electrically triggered sensorimotor potentials into a motor output at the ventral roots (system level), which determines the muscle force.

Motor behaviors are determined by how different synaptic inputs to motoneurons are integrated to produce the firing patterns of spinal motoneurons, and by how these individual motoneuron outputs are integrated into system output (muscle force). In the spinal cord, three excitatory sources determine the firing pattern of motoneurons: Descending motor commands from supraspinal structures (referred to as ‘*motor inputs*’), segmental sensory inputs from the periphery (referred to as ‘*sensory inputs*’), and local interneuronal inputs within the spinal cord (Carp and Wolpaw, 2010). Both the sensory and descending motor fibers form mono- and/or oligo-synaptic connections with motoneurons in the spinal cord (Eccles, 1946; Brown and Fyffe, 1981; Riddle et al., 2009; Liang et al., 2014; Witham et al., 2016). Therefore, the generation of an appropriate motor output for a given motor task is determined by the integration of sensory and motor inputs in the spinal cord and their ability to further engage local premotor interneurons (sensorimotor integration) (Rossignol et al., 2006; Kim et al., 2017).

As premotor interneurons fire repetitively, their synapses on motoneurons undergo typical short-term plasticity alterations (i.e., short-term facilitation or depression). Such plasticity is common to all neurons in the nervous system (Zucker and Regehr, 2002; Regehr, 2012). With successive electrical stimuli, some synaptic inputs to the target motoneuron become progressively smaller (i.e., synaptic depression), whereas others become progressively larger (i.e., synaptic facilitation). These forms of plasticity can be potentially regulated by the level of monoaminergic neuromodulators in the spinal cord. Neuromodulators such as serotonin and noradrenaline have powerful effects on the dynamics of synapses, and can even convert short-term synaptic depression to facilitation (Bevan and Parker, 2004; Barriere et al., 2008; Zhao et al., 2011; Nadim and Bucher, 2014). Presynaptically, Ca^2+^ influx and vesicular release probability are targets for neuromodulators (Logsdon et al., 2006; Higley and Sabatini, 2010). Postsynaptic neuromodulation, on the other hand, can be achieved through controlling ionic conductances on the dendrites that amplify or suppress synaptic currents (Lee and Heckman, 2000; Heckman et al., 2004; Sun et al., 2005; Miles et al., 2007). Recent studies show that electrical stimulation of either dorsal root sensory inputs or local motor inputs to motoneurons results in variable forms of short-term plasticity (Barriere et al., 2008; Jiang et al., 2015). It is currently unknown how these forms of plasticity change in presence of monoaminergic neuromodulation. Nonetheless, these variable sensory and motor inputs integrate during normal movements and thus are needed after SCI in order to generate steady motor outputs.

We therefore hypothesized that combined electrical stimulation of sensory and motor inputs in the transected spinal cord yields higher and more stable motor output than that generated by either input separately. To test this hypothesis, we used a whole-tissue *ex vivo* spinal cord preparation from adult mice to study the synaptic, cellular, and system effects evoked by electrical stimulation at different amplitudes (1.5× and 10× threshold), frequencies (25 and 50 Hz), and neuromodulatory states (in presence or absence of a noradrenergic agonist). The synaptic (i.e., excitatory postsynaptic potentials, EPSPs), cellular (i.e., motoneuron action potentials, APs), and system (ventral roots compound APs, coAPs) responses were simultaneously recorded during stimulation. Stimulation protocols included the sensory input (‘S’ condition, via dorsal roots), motor input (‘M’ condition, via descending tracts), or both (‘S&M’ condition, via dorsal roots and descending tracts). Our results show (1) EPSPs and coAPs of the S input exhibited progressive depression, (2) EPSPs and coAPs of the M input exhibited progressive facilitation, and (3) their combined effect (S&M) generated higher EPSP and coAP amplitudes with steady profiles. This combined enhancement was magnified at higher neuromodulatory states. Additionally, while the sensory and motor EPSPs exhibited sublinear summation, coAPs exhibited supralinear summation. Computer simulations of our experimental data showed that the differing summation of sensory and motor inputs at the cellular and system levels is because the motoneurons’ firing threshold acts as an amplitude-selective filter. This transforms small EPSP amplitude changes into large coAP amplitude responses. The simulations also demonstrated that the integrated S&M EPSPs maintained motoneuron membrane potential above the firing threshold longer. This generated steady motor output with successive stimuli, which could not be achieved by either input separately. In sum, these data provide mechanistic insights into how and why combining sensory feedback with motor stimulation evokes strong muscle contractions and stable motor function in patients with SCI. These results can be leveraged into improved stimulation protocols, leading to improved restoration of movement and independence for SCI patients.

## Materials and Methods

### Animals

The fifty three male mice (B6SJL, Jackson laboratory, Bar Harbor, ME) were used in this study, 30–200 days old. The sacrocaudal spinal cord was surgically transected and isolated, maintained *in vitro*, and used to record single motoneuron behavior as well as ventral root response. This part of the adult spinal cord can be reliably maintained *in vitro* for several hours (Bennett et al., 2001; Jiang and Heckman, 2006). All surgical and experimental procedures in this study were carried out in accordance with the recommendations of Wright State University Animal Care and Use Committee. The protocol was approved by the Wright State University Animal Care and Use Committee.

### *Ex vivo* Whole Tissue Spinal Cord Preparation

The procedures for surgical isolation of the sacrocaudal spinal cord has been previously described (Mahrous and Elbasiouny, 2017). Briefly, the animal was first deeply anesthetized using urethane (≥ 0.18 g/100 g, injected intraperitoneally). When the animal no longer responded to toe pinching, it was placed in a dissection dish and supplied with carbogen (95% O_2_/5% CO_2_) through a face mask. The spinal cord was exposed by means of dorsal laminectomy and longitudinal incision of the dura mater. The cord was transected around L4 segment and the caudal part with the attached roots was transferred to a dissection dish full of oxygenated modified artificial cerebrospinal fluid (mACSF, see below). The ventral and dorsal roots were separated and the cord was transected at the lumbosacral enlargement (L6). The cord was then pinned with the ventral side upward in a recording chamber and perfused with oxygenated normal artificial cerebrospinal fluid (nACSF, see below) at a rate of 2.5–3 ml/min. The dorsal and ventral roots were mounted on bipolar wire electrodes and covered with petroleum jelly to prevent drying. All experiments were performed at room temperature (∼21°C).

### Electrophysiological Recordings

#### Ventral Root Recording

Ventral roots were connected to a differential amplifier (Kinetic Software, GA) with 1000× gain and 300 Hz – 3 KHz bandwidth filter. We performed most of our recordings at the S4 segments where the responses were most stable and highest in amplitude (see below in ‘sensory and motor inputs’). During the first 30–40 min in the recording chamber, the ventral root response to dorsal root stimulation steadily increased in amplitude. Hence, the cord was allowed to recover for nearly 1 h before any recordings were started. The ventral root responses to trains of electrical stimulation were quantified as the peak-to-peak measurements of its compound action potentials (CoAPs).

#### Single Motoneuron Recordings

Using sharp intracellular glass microelectrodes, single motoneurons were recorded in the isolated whole tissue. These glass electrodes were pulled using a micropipette puller (P97, Sutter instruments, CA), and filled with 3M potassium acetate and 100 mM KCl and had a resistance of 25–40 MΩ. The microelectrodes were advanced into the ventral horn using a micro-positioner (2660, Kopf instruments, CA). Motoneurons were identified by antidromic stimulation of the ventral root and were accepted for recording when the resting membrane potential was below −60 mV and the antidromic spike was ≥ 60 mV. The Na^+^ channel blocker, QX-314 (50–100 mM), was used in the internal electrode solution to inhibit action potential generation, so that the exact amplitude of the synaptic potentials can be determined. The amplitudes of synaptic potentials during a train of stimulation were measured as the voltage change at the peak of the EPSP relative to the baseline resting membrane potential before the stimulation train. Intracellular recordings were performed using an Axoclamp 2B amplifier (Molecular Devices, CA) running in bridge or discontinuous current clamp (DCC) mode and low-pass filtered at 3 kHz.

The outputs of both the intracellular and extracellular amplifiers were digitized using Power 1401-3 data acquisition interface (CED, United Kingdom) at 10–20 kHz. Data were acquired into a computer controlled by Spike2 software (version 8.06, CED) and stored for offline analysis.

### Sensory and Motor Inputs

#### Sensory Inputs

**FIGURE 1:**
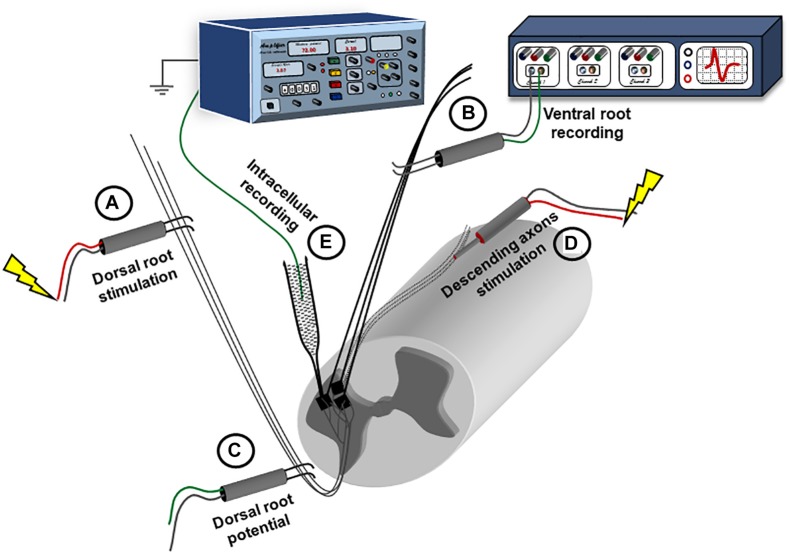
Experimental setup for recording synaptic responses to sensory and descending stimulation. The spinal cord is placed ventral side up in a perfusion chamber. Ventral and dorsal roots are mounted on bipolar wire electrodes above the solution level, and covered with petroleum jelly. The dorsal roots **(A)** are connected to a stimulator; while the ventral roots **(B)** are connected to a multichannel extracellular amplifier for recording. Another extracellular recording electrode is placed on the dorsal root **(C)** more proximally and connected to the extracellular amplifier to record the dorsal root volley. A concentric electrode **(D)** is placed on the ventral side to stimulate the descending axons. Intracellular recording is performed using sharp electrodes **(E)** advanced through the ventral surface.

The sensory inputs (S) were induced by electrical stimulation of the dorsal roots. Dorsal roots were connected through bipolar wire electrodes (Figure 1, electrode ‘A’) to a stimulator (Isoflex, AMPI), and stimulated with 0.1 ms pulses at either 1.5 or 10 times threshold (1.5×T or 10×T). The threshold for dorsal root stimulation was defined as the smallest amount of current delivered to the dorsal root to produce a minimal response (compound action potential, coAP) in the ventral roots, and ranged from 1.5 to 6 μA. In some experiments, another bipolar electrode was placed on the dorsal root more proximally to record the root potential. The response to dorsal root stimulation was most consistent and stable at the lower sacral as well as the caudal ventral roots (S3 to Co2).

#### Motor Inputs

The motor inputs (M) were induced by electrical stimulation of the spinal cord tissue (Figure 1, electrode ‘D’). A concentric bipolar electrode (0.125 mm stainless steel contact diameter insulated by Teflon from a stainless steel tube 0.2 ID/0.35 OD) was placed on the ventral surface close to the midline at the L6 segment (Jiang et al., 2015). Brief electrical pulses (0.1 ms) were delivered through the concentric electrode at 1.5×T or 10×T. The threshold was defined as the smallest amount of current, delivered through the concentric electrode, which produces a response in the ipsilateral ventral roots, and ranged from 100 to 200 μA. The largest response to motor stimulation was at the S1 segment and the smallest was at the caudal segments. Therefore, we conducted our recordings at S4 where the responses to both sensory and motor inputs were stable.

The response to each synaptic input was recorded separately, and then both pathways were stimulated simultaneously to study integration. To induce a higher neuromodulatory state, the α_1_-adrenergic receptor agonist, methoxamine (10 μM), was added to the recording solution. After 10 min of continuous exposure, the electrical stimulation paradigm was repeated.

#### Effective Synaptic Currents

To measure the effective synaptic currents generated by the sensory and motor inputs in Figure 7, we modified the methodology described in Heckman and Binder (1988). We used the voltage change at the fifth pulse as the steady state input in our protocol. The protocol was repeated with different values of injected current through the intracellular microelectrode. The relationship established between the injected current and the voltage change was then used to estimate the effective synaptic currents, and the slopes were compared to test if the synaptic inputs changed the input resistance of the cell.

### Physiological Solutions

#### Normal Artificial Cerebrospinal Fluid

The nACSF was formed of the following (in mM): 128 NaCl, 3 KCl, 1.5 MgSO_4_, 1 NaH_2_PO_4_, 2.5 CaCl_2_, 22 NaHCO_3_, and 12 glucose. The osmolarity of the solution was ∼295 mOsm, and the pH was 7.35–7.4 when aerated with carbogen (95% O_2_ and 5% CO_2_).

#### Modified Artificial Cerebrospinal Fluid

The mACSF contained the following (in mM): 118 NaCl, 3 KCl, 1.3 MgSO_4_, 5 MgCl_2_, 1.4 NaH_2_PO_4_, 1.5 CaCl_2_, 24 NaHCO_3_, and 25 glucose. This high Mg^2+^/low Ca^2+^ solution decreases the activity in the cord during dissection. The osmolarity of the solution was ∼310 mOsm, and the pH was 7.35–7.4 when aerated with carbogen (95% O_2_ and 5% CO_2_).

### Drugs and Chemicals

N-(2,6-Dimethylphenylcarbamoylmethyl) triethyl ammonium bromide (QX-314), a membrane-impermeable blocker of the voltage-gated Na^+^ channels; strychnine (STR), a blocker of the glycine receptors; picrotoxin (PTX), a blocker of the GABA_A_ receptors; methoxamine, an agonist of the alpha-1 adrenergic receptors. All of the drugs were purchased from Sigma (St. Louis, MO, United States), and all of the chemical components of the physiological solutions were purchased from Thermo Fisher Scientific (Waltham, MA, United States).

### Computational Models

#### Computer Model of the Motor Pool

To simulate the response of spinal motoneurons to electrically evoked sensory and motor inputs, we employed a multiscale, high-fidelity computer model of the alpha-motoneuron pool, which is described in full detail in Jiang et al. (2015). This model is based on 3D reconstructed motoneuron anatomy data and bridges the synaptic, cellular, and system scales. Motoneurons with reconstructed morphologies, as opposed to simplified computer models of motoneurons, were employed because the simplification of dendritic morphology has been shown to generate large errors in simulating motoneuron firing behaviors and active properties (Elbasiouny, 2014). The motor pool model consisted of 50 cells and was implemented using the NEURON simulation environment (Hines and Carnevale, 1997). The model of individual cells was based on that developed for alpha motoneurons by Elbasiouny et al. (2005), which incorporated realistic alpha motoneuron morphology, realistic dendritic distribution of synaptic inputs, and somatic and dendritic active conductances. This cell model was used because it has been highly optimized to reproduce multiple electrophysiological datasets of spinal motoneurons obtained under different recording conditions (current- and voltage-clamp, motoneuron activation via synaptic inputs and current injection) (Elbasiouny et al., 2005, 2006). The somatic active conductances included the fast Na^+^ and delayed rectified K^+^ channels (which mediate the AP spike), and the Ca^2+^-activated K^+^ channels and N-type Ca^2+^ channels (which mediate the afterhyperpolarization, AHP). The dendritic active conductances included the low voltage-activated L-type Ca^2+^ channels (which mediate Ca^2+^ persistent inward current, Ca^2+^ PIC). The passive and active properties of the Elbasiouny et al. (2005) model were varied to match the electrical properties of sacral motoneurons of different types (i.e., slow, fatigue-resistant, and fast-fatigable types) in order to more accurately represent the motoneuron pool.

Each individual motoneuron cell model was driven by two sources of synapses: sensory and motor synapses. The distribution of excitatory and inhibitory sensory and motor synapses was uniform on the motoneuron dendrites and their conductances were the same across all cells in the pool. This assumption is supported by the equal synaptic input limb motoneurons receive from inhibitory Renshaw and Ia-reciprocal inhibitions and the small variability in amplitude of inputs motoneurons receive from excitatory Ia-afferents and vestibulospinal input (Powers and Binder, 2001). Following the methodology of Jiang et al. (2015), the excitatory and inhibitory conductances of the sensory and motor synapses were calculated from the experimental data and adjusted in order to reproduce our ventral root recordings before and after administration of STR and PTX (Table 1 shows the synaptic conductances for the sensory and motor inputs). To simulate the effects of electrical stimulation of the sensory and/or motor inputs, the sensory and motor synapses were activated synchronously and repeatedly at frequencies comparable to the stimulation frequencies tested in our experiments (i.e., 25 and 50 Hz). Accordingly, the synaptic conductances in the model accounted for the strength of the synapses on motoneurons and the probability of neurotransmitter release at that stimulation frequency. Following the methodology of Jiang et al. (2015), the model also incorporated the depolarization in membrane potential between pulses for the sensory and motor inputs.

**TABLE 1:** Synaptic conductances of the sensory and motor inputs in the simulations.

**Pulse #**	**Synaptic conductances (μS)**			
	**Sensory input**		**Motor input**	
	**Excitatory**	**Inhibitory**	**Excitatory**	**Inhibitory**
P1	0.016	0.014	0.009	0.0022
P2	0.00976	0.0063	0.00954	0.0022
P3	0.0096	0.00588	0.009504	0.00187
P4	0.0104	0.00868	0.00954	0.00242
P5	0.01008	0.00868	0.009765	0.00308

To mimic our EPSP recordings in presence of QX-314 in the micropipette, we measured EPSPs in simulations when Na^+^ channels were blocked.

#### Driving Force Simulations

To investigate the effects of changing the driving force on synaptic integration (Figure 8), we used an FR cell from the pool model to run these simulations. In these simulations, the somatic voltage-gated conductances were removed from the model to isolate the synaptic effects. The sensory and motor synapses were distributed uniformly over the dendrites (Figure 8A). The onset and time to peak for each synapse were matched to the experimental data (Table 2) for the sensory and motor inputs. The synapses were activated using a single pulse, and the maximum conductances for each synapse were weighted based on the relative amplitudes of the first response generated by each input at 1.5×T intensity in the experimental data. All model parameters were kept unchanged during each run except for the reversal potential of the synapses, which was varied to change the driving force (resting membrane potential kept at −70 mV). Sensory and motor synaptic responses in our preparation were eliminated by DNQX (blocker of AMPARs, not shown) indicating that these synapses are glutamatergic. Therefore, sensory and motor synapses were given the same equilibrium potential during each run.

**TABLE 2:** Different timing parameters for the synaptic potentials of the sensory and motor inputs.

**Time parameters (Mean ± *SD*, milliseconds)**	**Sensory input**	**Motor input**	**Number, *p*-value**
Delay from trigger	1.97 ± 0.54	1.85 ± 0.32	*N* = 11, *p* = 0.46
Time to peak (T_Peak_)	1.69 ± 0.58	2.14 ± 0.59	*N* = 11, *p* = 0.04
Half decay time (T_1/2_)	4.3 ± 1.86	5.71 ± 1.78	*N* = 11, *p* = 0.01

### Statistical Analysis

The statistical analyses were performed using GraphPad Prism software (La Jolla, CA, Version 7.01). Repeated-measures one-way ANOVA was used to test changes in the amplitude of coAPs and EPSPs at different stimulation pulses to a specific pathway (i.e., sensory, motor, or sensorimotor). When the response at each pulse was compared between two different pathways, or before and after drug treatment, a repeated-measures two-way ANOVA was used. For *post hoc* analyses, Tukey test was used with two-way ANOVA and Dunnett test was used with one-way ANOVA. *T*-test was used to compare the delay, time to peak, and decay time of EPSPs of different pathways. A *p*-value of < 0.05 was considered significant for all tests.

## Results

In this study, the whole-tissue adult sacrocaudal spinal cord was used to study the plasticity, integration, and neuromodulation of electrically evoked sensory and motor synaptic inputs generated in spinal motoneurons. The sensory input was activated by electrical stimulation of the ipsilateral dorsal roots, which generates a response that contains a prominent monosynaptic component, presumably from the Ia muscle afferents. On the other hand, the motor input was activated via surface electrical stimulation of the ventrolateral funiculus of the rostral end of the sacrocaudal preparation. This stimulation activates local interneurons in addition to the remaining axons of descending tracts, primarily the lateral vestibulospinal tract (LVST, see section “Discussion” for more details). The synaptic, cellular, and system responses to electrical stimulation were recorded as EPSPs and somatic APs measured from single motoneurons using intracellular sharp electrodes and coAPs measured from the ventral roots using extracellular wire electrodes, respectively (Figure 1).

Short trains of electrical stimuli (five pulses) of low intensity (1.5×T, T is threshold of each pathway) or high intensity (10×T) at physiological frequencies (25 Hz or 50 Hz) were delivered to either the dorsal roots (sensory input, S), the remaining descending axons (motor input, M), or both simultaneously (sensorimotor input, S&M). Threshold was determined separately for the S and M inputs, and was identified as the minimum stimulation intensity needed to activate that particular pathway to evoke the smallest observable response in the ventral roots.

### Electrically Evoked Sensory Inputs Exhibit Depression With a Train of Stimulation

**FIGURE 2:**
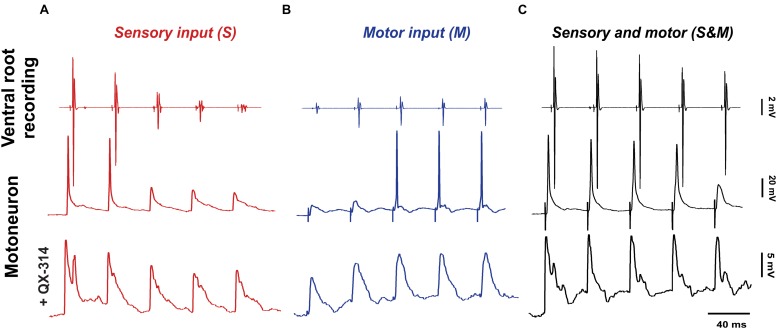
Adapting sensory and motor inputs generate a steady motor output. The response to electrical stimulation (5 pulses at 25 Hz) of the dorsal roots (**A**, red), descending axons (**B**, blue) or both of them simultaneously (**C**, black). The top panel shows compound action potentials (coAPs) recorded in the ventral roots, while the middle and bottom panels show spikes and synaptic potentials (EPSPs) in single motoneurons with or without the Na^+^ channel blocker QX-314 in the internal electrode solution, respectively.

Electrical stimulation of the dorsal roots (S input), at both intensities (1.5×T and 10×T) and frequencies (25 Hz and 50 Hz), generated coAPs in the ventral roots that became progressively smaller with successive pulses of stimulation (Figure 2A, top panel). This pattern was consistent among different roots and different animals at intensities of 1.5×T (red bars labeled ‘S’ in Figure 4A, and Supplementary Figure 1A) and 10×T (red bars labeled ‘S’ in Figure 5A, and Supplementary Figure 2A); *n* = 15, repeated-measures one-way ANOVA, *p* < 0.0001 for all intensity/frequency combinations.

When sensory synaptic responses were recorded intracellularly in single motoneurons, the cells initially fired action potentials, but later failed with subsequent pulses (Figure 2A, middle panel). To measure the amplitude of sensory EPSPs, QX-314 (a blocker of the voltage-gated Na^+^ channels) was added to the internal microelectrode solution in order to block cell spiking (Figure 2A, bottom panel). Similar to coAPs, sensory EPSPs exhibited progressive depression in response to a train of stimulation to the dorsal root at the tested frequencies and intensities (red bars of Figures 4B, 5B and Supplementary Figures 1B, 2B, *n* = 12/each, repeated-measures one-way ANOVA, *p* ≤ 0.005 for all intensity/frequency combinations). This gradual depression occurred in spite of the membrane depolarization (1 ± 0.17 mV at 1.5×T and 1.76 ± 0.3 mV at 10×T) during the pulse train (Figure 2A bottom panel and Supplementary Figure 3A). Importantly, the depression seen in the coAPs’ amplitude was much more pronounced compared to the depression seen in EPSPs (in Figure 4 and Supplementary Figure 1, compare red bars labeled ‘S’ in A and B of the same figure). For instance, by the 5th pulse of stimulation, the amplitude of coAPs was reduced on average by 95%, whereas the amplitude of corresponding EPSPs was reduced on average by only 33%. This indicates a disproportion between the cellular (EPSP data) and system (coAP data) plasticity in the sensory input to motoneurons.

### Plasticity of the Sensory Inputs Is Partially Due to Short-Term Synaptic Depression

The depression seen in the response to sensory inputs could result from one or more of several factors: (1) sensory axons fail to fire at the stimulation frequencies used, (2) activation of polysynaptic inhibitory pathways in the cord affects later pulses than earlier ones, and/or (3) depression of synaptic transmission caused by depletion of vesicular neurotransmitter release at the terminal. The following experiments aimed to separate and quantify these factors.

**FIGURE 3:**
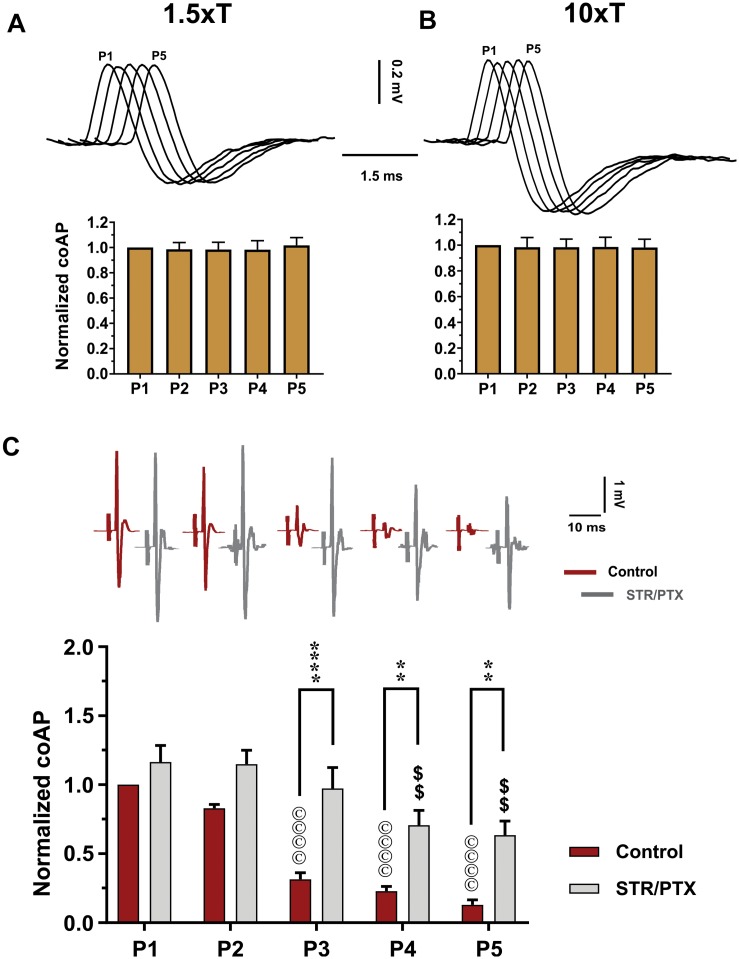
Depression of the sensory inputs is partially due to activation of inhibitory pathways, not stimulation failure. The dorsal root potentials recorded in response to electrical stimulation of the roots’ distal end at 1.5×T **(A)** and 10×T **(B)** do not show any adaptation. Top: Example dorsal root potentials recorded in the same dorsal root at different intensities. Bottom: Summary of responses from multiple experiments. The root potentials were normalized to the amplitude of the first response in each experiment. Data show no stimulation failure during a 5-pulse train at 25 HZ (*n* = 12, repeated-measures one-way ANOVA, *p* = 0.29 and *p* = 0.66, respectively – error bars represent *SD*). **(C)** CoAPs recorded in the ventral roots exhibit gradual depression in response to a train of stimulation to the dorsal root. This depression is partially alleviated when synaptic inhibition is blocked by strychnine (STR) and picrotoxin (PTX; *n* = 8, repeated-measures two-way ANOVA, *p* = 0.0001). Top: Example coAPs recorded under normal conditions (black trace) and in presence of synaptic inhibition blockers in the same ventral root (gray trace). Bottom: summary of coAPs’ amplitudes in presence and absence of STR/PTX. The coAps were normalized to the amplitude at the 1st pulse under control conditions. *The symbol* ‘©’ *denotes significant difference from control P1 response, and ‘$’ denotes significant difference from P1 response in presence of STR/PTX.*

First, to test if the observed depression is due to the failure of sensory axons in conducting APs at the stimulating frequencies, we used a recording electrode on the dorsal root (labeled ‘C’ in Figure 1) to record the dorsal root potential, which has a magnitude proportional to the number of stimulated sensory axons. Repeated-measures one-way ANOVA showed that dorsal root potentials at all pulses were not different when stimulated at 1.5×T (Figure 3A, *n* = 12, *p* = 0.3) or 10×T (Figure 3B, *n* = 12, *p* = 0.7). This indicates that similar number of sensory axons were consistently stimulated and recruited by each of the five pulses. Therefore, the depression observed in sensory coAPs and EPSPs is not due to failure of stimulation. Interestingly, when the stimulation intensity was increased to 100×T, no further increase in the dorsal root potential amplitude was seen relative to 10×T, indicating that 10×T recruited the maximum number of sensory axons.

Second, to test the contribution of inhibitory pathways to the measured sensory depression, strychnine and picrotoxin (selective blockers of glycine and GABA) were administered to block inhibitory synaptic transmission. In presence of strychnine and picrotoxin, the sensory input showed less depression (Figure 3C, *n* = 8, repeated-measures two-way ANOVA, *p* < 0.001). For instance, the amplitude of coAPs in the 4th and 5th pulses (when polysynaptic pathways have been stimulated more relative to the early pulses) was reduced by 37–42% after administration of strychnine and picrotoxin, as opposed to 80–90% depression before their administration (Figure 3C, *p* < 0.01). These data indicate that about 50–60% of the sensory depression is mediated by activation of polysynaptic inhibitory pathways. Thus, the remaining decline can be attributed to use-dependent depression at the presynaptic terminals.

### Electrically Evoked Motor Inputs Exhibit Facilitation With a Train of Stimulation

**FIGURE 4:**
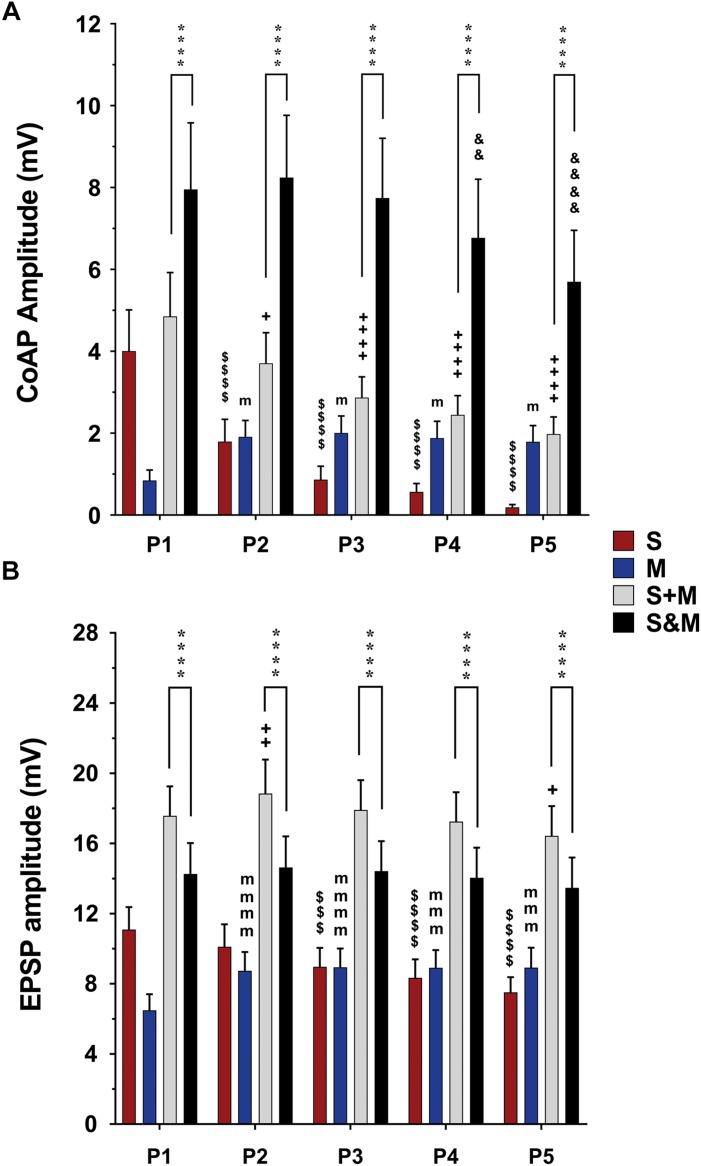
Summary of the responses to individual and integrated sensory and motor inputs at 1.5×T/25 Hz stimulation. Summary of responses in the ventral roots **(A)** and single motoneurons **(B)** to 5-pulse electrical stimulation (P1–P5) at a frequency of 25 Hz delivered to the dorsal roots (sensory inputs, ‘*S*’), descending fibers (motor inputs, ’*M*’), or both (sensorimotor integrated inputs, ‘*S&M*’). **(A)** In the ventral roots, coAPs generated by the sensory inputs exhibit depression while those generated by the motor inputs (M) exhibit facilitation. When compared to the linear summation of the two inputs (S + M), the simultaneous activation of the two inputs (S&M) results in a steadier motor output, and supralinear summation of the coAPs (S&M > S + M), *n* = 15. **(B)** At the cellular level, the EPSPs generated by each individual pathway follow the same adaptation pattern as the coAP, though less dramatically. The integration of the two inputs results in non-adapting synaptic potentials, and sublinear summation of the EPSPs (S + M > S&M), *n* = 12. Data represented as the mean ± SEM. Repeated-measures one-way ANOVA was used to study the pattern of adaptation of each input. Repeated-measures two-way ANOVA was used to test the type of integration (S&M vs. S + M). The symbol ‘$’ denotes significant difference from S P1, ‘m’ denotes significant difference from the M P1, ‘ + ’ denotes significant difference from S + M P1, ‘&’ denotes significant difference from S&M P1, and ‘^*^’ denotes significant difference between S&M and S + M.

**FIGURE 5:**
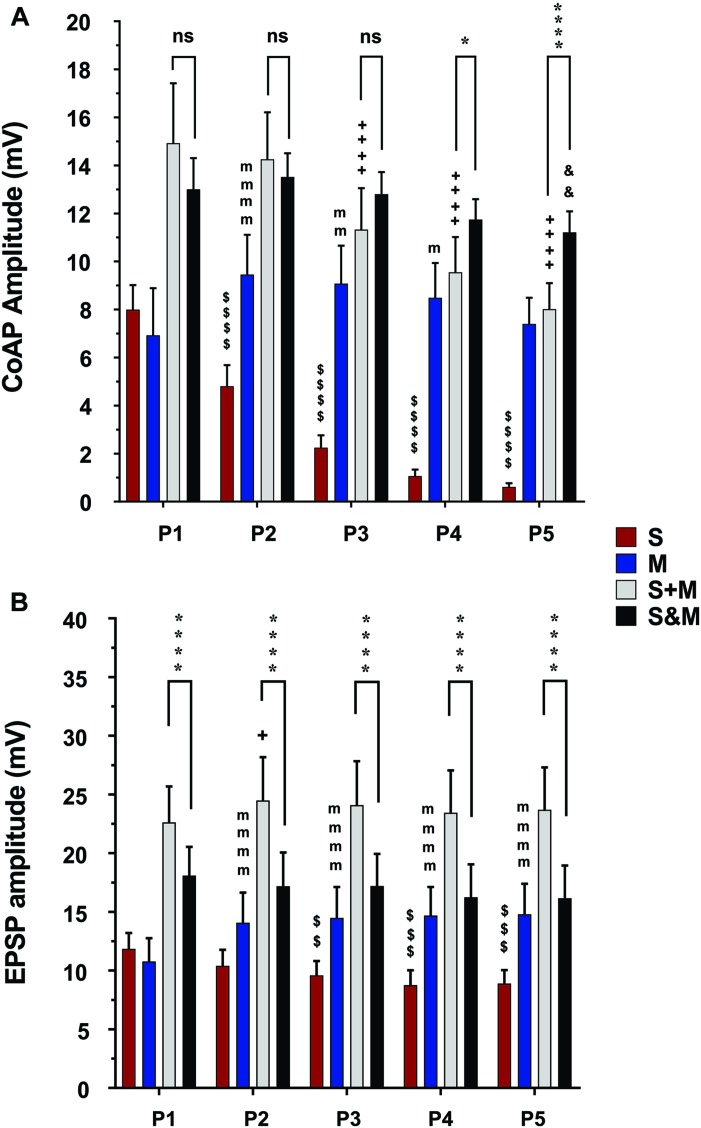
Summary of the responses to individual and integrated sensory and motor inputs at 10×T/25Hz stimulation. Summary of responses to 10×T/25 Hz electrical stimulation of the sensory and descending pathways (The layout is similar to Figure 4). **(A)** In the ventral roots, the responses follow the same adaptation patterns as the lower stimulation intensity. The integration of the two inputs results in a steadier motor output and linear summation of the coAPs, except at P4 and P5, where it becomes supralinear (S&M ≥ S + M), *n* = 15. **(B)** In motoneurons, integration of the two inputs results in non-adapting synaptic potentials (S&M) and sublinear summation (S + M > S&M), *n* = 12. Data representation, statistical analysis, and significance symbols are the same as in Figure 4.

To activate the motor input, electrical stimulation was delivered to the ventral surface of the spinal cord below the lumbosacral enlargement next to the midline. A similar protocol of 5-pulse trains at intensities of 1.5×T or 10×T and frequencies of 25 or 50 Hz was used. Electrical stimulation evoked coAPs in the ventral roots that gradually increased in amplitude (Figure 2B, top panel). Again, this pattern was consistent among different roots and different animals at intensities of 1.5×T (‘M’ labeled bars in Figure 4A and Supplementary Figure 1A) and 10×T (blue bars labeled ‘M’ in Figure 5A and Supplementary Figure 2A); *n* = 15/each, repeated-measures one-way ANOVA, *p* < 0.01 for all intensity/frequency combinations.

In single motoneurons, motor EPSPs evoked by a train of stimulation gradually increased in amplitude, which sometimes resulted in generation of action potentials with the later pulses (Figure 2B, middle panel). When action potentials were blocked by QX-314 in the microelectrode (Figure 2B, bottom panel), motor EPSPs showed similar patterns of facilitation at the tested frequencies and intensities (blue bars labeled ‘M’ in Figures 4B, 5B and Supplementary Figures 1B, 2B, *n* = 12/each, repeated-measures one-way ANOVA, *p* ≤ 0.008 for all intensity/frequency combinations). The membrane potential of motoneurons exhibited consistent progressive depolarization during motor stimulation (Figure 2B and Supplementary Figure 3) which contributes to the facilitation of the synaptic potentials.

The degree of motor facilitation was more profound in the coAPs compared to EPSP synaptic potentials (in Figure 4 and Supplementary Figure 1, compare blue bars labeled ‘M’ in A and B of the same figure). The coAPs at the second to the fifth pulse increased by more than 110%, compared to only a 34% increase in the EPSPs’ amplitudes. This indicates disproportionality between the cellular and system plasticity, similar to that observed in the sensory input.

### Contrasting Summation of Electrically Evoked Synaptic Effects Between the Synaptic and System Levels

The data above show that electrically evoked sensory and motor inputs to motoneurons have different synaptic plasticity patterns in response to a train of electrical stimuli. We then investigated the characteristics (amplitude and profile) of the motor output generated from concurrent activation of both inputs (S&M). When dorsal roots and descending fibers were stimulated simultaneously, the S&M coAPs at the ventral roots exhibited less adaptation and had higher amplitudes than those generated from either input separately (Figure 2C, top panel). This pattern was consistent among different roots and different animals at intensities of 1.5×T (black bars labeled ‘S&M’ in Figure 4A and Supplementary Figure 1A) and 10×T (black bars labeled ‘S&M’ in Figure 5A and Supplementary Figure 2A). For instance, there was no change in S&M amplitudes between the first three pulses (Figure 4A). Relative to the 1st pulse response, the 4th and 5th S&M pulses showed an average decline of only 15 and 28%, respectively, compared to an 86 and 95% decrease in the S coAPs; to a 122 and 111% increase in the M coAPs; and to the linear summation of individual S and M inputs (termed ‘S + M’), which showed a 50 and 59% decrease in the coAPs. This shows that simultaneous activation of S&M inputs generated steadier coAPs with a train of stimulation.

At the synaptic level, the S&M EPSPs also exhibited a non-adapting pattern: Their amplitudes did not change at any pulse for 1.5×T and 10×T intensities [Figure 2C, bottom panel and black bars labeled ‘S&M’ in Figure 4B (*p* = 0.38) and Supplementary Figure 1B (*p* = 0.44) for 1.5×T and Figure 5B (*p* = 0.54) and Supplementary Figure 2B (*p* = 0.77) for 10×T]. This is different from the separate sensory or motor EPSPs, which each exhibited about a one-third decrease or increase, respectively, by the 5th pulse. This non-adapting pattern is likely because the facilitation generated from the motor input balances the depression generated from the sensory input, leading to steady EPSPs. This is also evident in the changes of the baseline membrane potential during stimulation. The progressive depolarization of the membrane during stimulation seems to be mainly driven by the motor pathway (Figure 2C and Supplementary Figure 3). Collectively, the data indicate that combining sensory and motor inputs generates a steadier motor output at the system level.

To quantify the integration of inputs, we compared the amplitudes of coAPs resulting from simultaneous stimulation of both pathways (referred to as ‘S&M’) to the linear sum of amplitudes of separate S and M coAPs (referred to as ‘S + M’). At the system level, the amplitudes of S&M coAPs were significantly larger than those of S + M coAPs at all five pulses at 1.5×T (*n* = 15, repeated-measures two-way ANOVA, *p* < 0.001), indicating supralinear summation of the responses at the system level (Figure 4A and Supplementary Figure 1A). With 10×T stimulation, supralinear summation was observed only at the last two pulses at 25 Hz (Figure 5A), but not at 50 Hz (Supplementary Figure 2A). At the synaptic level, S&M EPSPs summation pattern, on the other hand, was totally opposite – in which EPSPs exhibited sublinear summation (Figure 4B, S&M < S + M, *n* = 12, *p* = 0.003). These data indicate a discrepancy in input summation between the synaptic and system levels. Importantly, at all the tested stimulation frequencies/intensities, simultaneous activation of both inputs (S&M) evoked stronger and more stable motor output than the linear summation of individual inputs (S + M). Together, combining sensory and motor inputs generates higher as well as steadier motor output at the synaptic and system levels.

To this point, our results show that: (1) The S input exhibits depression at the synaptic (EPSP data) and system (coAP data) levels; (2) the M input exhibits facilitation at the synaptic and system levels; (3) there is a disproportion in the magnitude of induced plasticity between the synaptic and system levels for both inputs, in that plasticity is more profound at the system level than at the synaptic level; (4) simultaneous activation of both inputs (S&M condition) generates a stronger and steadier (i.e., less adaptive) responses at the synaptic and system levels; and (5) there is a discrepancy in summation of S and M inputs between the synaptic and system levels, in that EPSPs summate sublinearly, whereas coAPs summate supralinearly.

### Computer Simulations to Examine the Discrepancy in Summation and Disproportionality in Plasticity Between the Synaptic and System Levels

Our experimental data revealed that sensorimotor inputs (S&M) always summate supralinearly at the system level (Figures 4A, 5A) but always summate sublinearly at the synaptic level (Figures 4B, 5B). Additionally, the magnitude of depression in the sensory input (or the magnitude of facilitation in the motor input) was more pronounced at the system level than at the cellular level (compare Figures 4A, 5A to Figures 4B, 5B, respectively). To examine this discrepancy in summation and disproportion in plasticity of sensory and motor inputs between the synaptic and system levels, we employed a multi-scale high-fidelity computational model of the spinal motor pool to investigate the process of transformation of synaptic inputs into cell firing. We tested the hypothesis that the motoneuron firing threshold acts as an amplitude-selective filter, resulting in the summation discrepancy and plasticity disproportionality phenomena.

**FIGURE 6:**
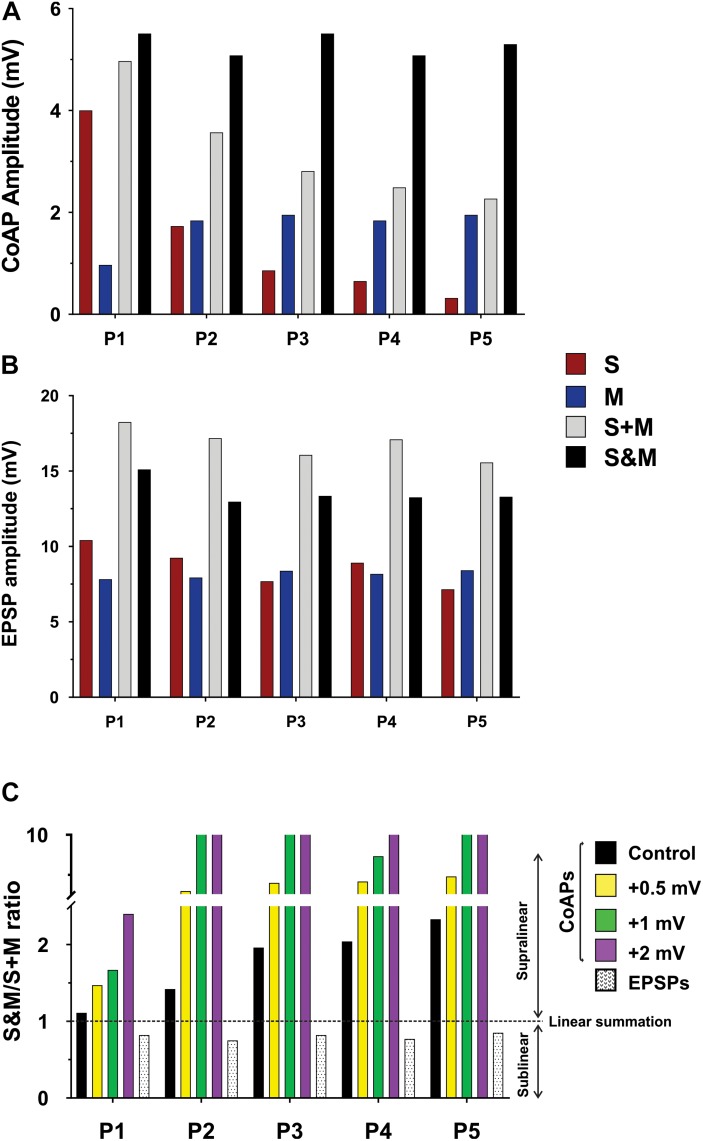
Computational model showing the effect of changing the firing threshold of individual motoneurons on the summation of sensory and motor inputs. Data obtained from a computational model of a motor pool (50 cells) which is stimulated using two separate uniformly-distributed synaptic inputs, and generates an output analogous to the experimental data. **(A)** The output of the motor pool (compare to Figure 4A). **(B)** The EPSP profile in a motoneuron (compare to Figure 4B). **(C)** The S&M/S + M response ratio of the pool output is dramatically increased by minor shifts of the cells’ firing threshold to more depolarized potentials. The summation of the EPSPs was sublinear and its magnitude was similar at all firing threshold levels, thus we show it at one level here.

We used the computational model of the spinal motor pool published in Jiang et al. (2015) to simulate our experimental recordings on the activation of the motoneuron pool (50 cells in the pool model) with 5 pulses at 1.5×T, via the sensory, motor, or both inputs. Two sets of synapses were uniformly distributed over the dendrites of each cell in the pool model: One set of synapses was used to represent the sensory input to each cell and another to represent the motor input to each cell. The synaptic conductances of each input were adjusted to simulate the amplitudes of coAPs and EPSPs as generated from the separate stimulation of the sensory or motor input at 25 Hz (Table 1 shows the synaptic conductances for the sensory and motor inputs). The increases in membrane potential observed experimentally between pulses for the sensory and motor inputs were also incorporated in the model. In that way, the model reproduced the amplitude and profile of depression in coAPs and EPSPs of the sensory input (compare the 1st bars in Figures 4A,B to the 1st bars in Figures 6A,B, respectively), reproduced the amplitude and profile of facilitation in coAPs and EPSPs of the motor input (compare the 2nd bars in Figures 4A,B to the 2nd bars in Figures 6A,B, respectively), as well as reproduced the depolarization in membrane potential between pulses. Importantly, when both inputs were simultaneously activated, the pool model replicated all features of the S&M experimental recordings. Specifically, the model replicated the steady, non-adapting profile of coAPs and EPSPs of the S&M condition (compare the 4th bars in Figures 4A,B to the 4th bars in Figures 6A,B, respectively). The model also replicated the supralinear summation of S&M coAPs relative to S + M (compare the 3rd and 4th bars in Figure 6A), as well as replicated the sublinear summation of S&M EPSPs relative to S + M (compare the 3rd and 4th bars in Figure 6B). Additionally, the model simulated the increasing difference between the S + M and S&M coAPs over the 5 pulses (compare the differences between the 3rd and 4th bars over the 5 pulses in Figure 6A to those in Figure 4A), as well as simulated the relatively constant difference between the S + M and S&M EPSPs at all 5 pulses (compare the differences between the 3rd and 4th bars over the 5 pulses in Figure 6B to those in Figure 4B). In sum, the pool model has accurately simulated the discrepancy in summation and disproportionality in plasticity of S&M inputs observed experimentally between the synaptic and system levels. We focused next on explaining these two phenomena using the computational model.

To test our hypothesis on the effect of the motoneuron firing threshold, we varied the firing threshold of individual motoneurons in the pool model to different levels while measuring the amplitudes of the resulting S&M coAPs and EPSPs. Each level was compared to the amplitudes from separate inputs at that level (Figure 6C, the horizontal dotted line represents linear summation, S + M). Importantly, when the motoneuron firing threshold was depolarized, the magnitude of the coAP supralinear summation increased proportionally at all pulses (compare the difference between the horizontal dotted line and the first 4 bars over the five pulses in Figure 6C). However, the magnitude of the EPSP sublinear summation was similar at all threshold levels (compare the difference between the horizontal dotted line and the last bar over the five pulses in Figure 6C). In other words, EPSPs always summate sublinearly and independently from the cell firing threshold (see next section for more explanation), whereas the coAP summation depends on the firing threshold: the more depolarized the firing threshold, the larger the supralinear summation. It is striking that small depolarizing shifts in the firing threshold (on the order of 0.5 mV) resulted in such large summation differences (Figure 6C). This variation in the coAP summation, but fixed EPSPs summation causes the disproportionality in plasticity observed between the synaptic and system levels.

### Sublinear Summation of Electrically Evoked EPSPs Is Due to Changes in Driving Force

At all the tested frequencies and intensities, the summation of the sensorimotor EPSPs was sublinear. This effect could result from: (1) an increase in the effective cell conductance, and/or (2) a decrease in the driving force of the synaptic current. The following set of experiments and simulations examined these potential mechanisms.

**FIGURE 7:**
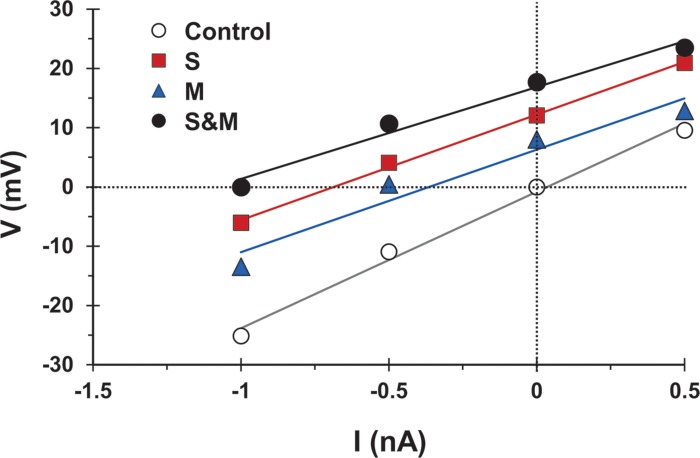
The activation of sensory and/or motor inputs does not cause a significant change in motoneuron conductance. Voltage measurements versus injected current for a motoneuron in response to injected current alone (open circles), and injected current combined with sensory input activation (S, squares), motor input activation (M, triangles), or combined sensorimotor input (S&M, filled circles). The solid lines indicate the best linear fit to the data points. Effective synaptic current for any of the synaptic inputs is equal in magnitude, but opposite in sign to the injected current at which V (injected + synaptic) = 0. The slopes of the fit lines are not different (Linear regression, *p* = 0.2) indicating that the activation of the synapses did not significantly change the cell input conductance, *n* = 4.

First, to test whether synaptic activation changed the motoneuron input conductance, we followed the method of Binder and Powers (1999) and measured the voltage response to injected current alone or combined with synaptic activation. We then compared the slope of the best linear fit of the voltage responses generated by injected current and synaptic activation (‘S’ condition, ‘M’ condition, or ‘S&M’ condition in Figure 7) to that of the injected current alone (‘control’ condition in Figure 7). Because the slope of the control line is determined by the cell input conductance, any change in the slope upon synaptic activation would indicate a change in the cell conductance (Binder and Powers, 1999). Because the slopes of the S, M, and S&M lines were not different from that of control (*n* = 4), this indicated that these synaptic inputs did not alter the cell input conductance and, therefore, did not contribute to the sublinear integration of sensory and motor EPSPs.

**FIGURE 8:**
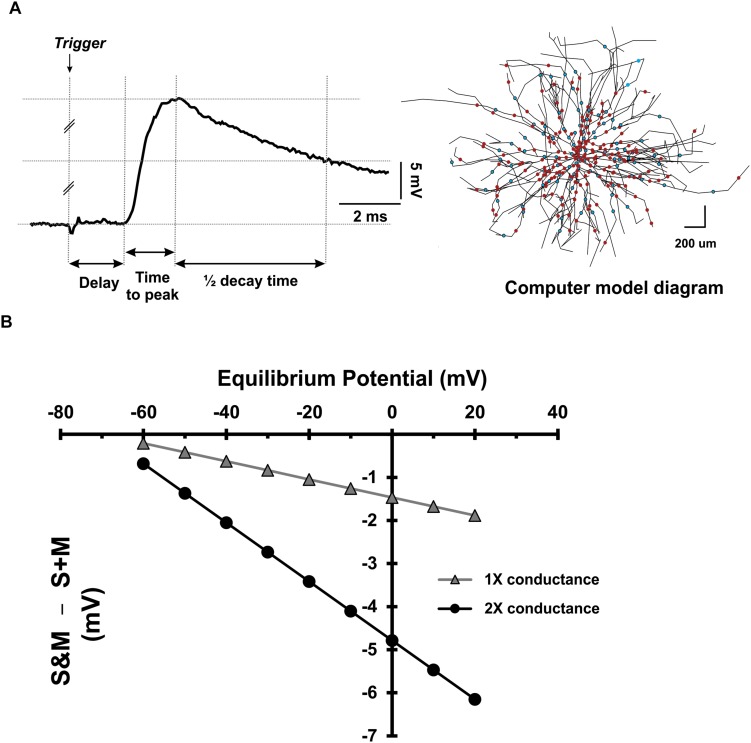
Dependence of EPSP summation on driving force. **(A)** *Left*: Measurement of different parameters of sensory and motor EPSPs to be used in a computer model. *Right*: a diagram of the motoneuron model used to investigate the effect of the driving force on sensory and motor EPSPs’ integration. The cell has detailed anatomy and generates sensory and motor EPSPs with the same parameters as experimental data. The dots indicate the location of the uniformly distributed synapses on the soma and dendrites (red for sensory synapses and blue for motor synapses). **(B)** The deviation of EPSP summation from linearity plotted as a function of the synapses’ equilibrium potential. Data collected using the model motoneuron in panel **(A)**.

Second, to investigate the effect of changing the driving force of the synapse, we measured different characteristics of the EPSPs as described in Figure 8A. The EPSPs generated from the S and M inputs had similar delays (*p* = 0.46, paired *t*-test), indicating that these EPSPs reached the motoneuron soma synchronously. However, the EPSPs generated by the M input took longer to reach their peak amplitude (*p* = 0.04, paired *t*-test) and longer to decay (*p* = 0.01, paired *t*-test) than those of the S input (experimental data summarized in Table 2). To test the effect of changing the synapse driving force on EPSPs summation, we simulated our EPSPs experimental recordings. Specifically, we incorporated the experimental data on S and M EPSPs (In Table 2) into one of the motoneurons in the computational pool model (Figure 8A, see methods for details). Also, the equilibrium potential of the synapses on that model cell was varied in order to test the effect of changing the synapse driving force on the difference between the integrated (S&M) EPSPs and the linear sum of individual (S + M) EPSPs (Figure 8B). As the driving force increased, the magnitude of sublinearity increased, and the gain of the relationship was determined by the conductances of the synapses (compare the gray and black traces in Figure 8B). Thus, strong synapses (i.e., large-amplitude EPSPs) would summate sublinearly more than weak synapses (i.e., small-amplitude EPSPs).

Taken together, the data show that sublinear summation of sensory and motor EPSPs at the synaptic level is caused by changes in local driving force, and not by increased input conductance of the cell.

### Effect of the Neuromodulatory State on Adaptation and Integration of Electrically Evoked Potentials

The level of neuromodulation sets the excitability level of both presynaptic and postsynaptic neurons in the spinal network and thus affects patterns of synaptic plasticity (Barriere et al., 2008; Nadim and Bucher, 2014). The *ex vivo* spinal cord preparation used in this study represents a relatively low neuromodulatory state (i.e., low excitability level) due to the absence of the neuromodulation normally provided via serotoninergic and noradrenergic descending inputs. It resembles, however, the clinical situation of complete SCI.

**FIGURE 9:**
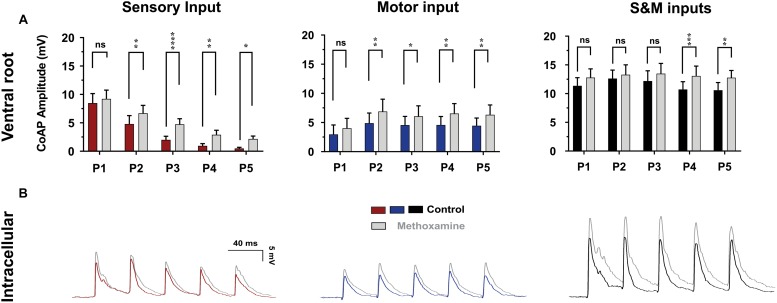
The effect of methoxamine on plasticity and integration of sensorimotor inputs. **(A)** The effect of methoxamine (10 μM) on the ventral root response to 10×T/25 Hz stimulation, showing separate and combined sensory and motor inputs. Methoxamine enhanced the response to both the sensory and motor inputs, and helped generate a more stable motor output. Data represented as the mean ± SEM. Repeated-measures two-way ANOVA was used to test the effect of methoxamine on each type of synaptic inputs, *n* = 6. **(B)** The application of methoxamine (10 μM) to the bath solution increases the amplitude and slows down the decay of the synaptic potentials, *n* = 7. The recording microelectrode contained QX-314 to prevent spiking.

Recent studies show that pharmacological neuromodulation improves the response to electrical stimulation after SCI in *in vivo* rodent studies (Courtine et al., 2009) as well as human clinical studies (Gerasimenko et al., 2015). To study the effect of enhanced neuromodulatory state on sensorimotor integration in the spinal cord, 10 μM of methoxamine, an α_1_-adrenergic receptor agonist, was applied (Lee and Heckman, 1999) and the ventral root coAPs at 10×T/25 Hz were recorded (Figure 9, *n* = 6). Using repeated measures two-way ANOVA, and Tukey *post hoc* test, the response at each pulse after adding methoxamine was compared to its corresponding one before treatment. Importantly, addition of methoxamine enhanced the coAP responses to the sensory (*p* < 0.001) and motor (*p* = 0.007) inputs. The S&M EPSPs became much larger in presence of methoxamine (Figure 9B), generating steadier coAPs that became larger at the last two pulses (*p* = 0.03).

Interestingly, methoxamine also prolonged the decay of EPSPs (compare the falling phases of the EPSPs before and after methoxamine in Figure 9B, *n* = 7), leading to elevated membrane depolarization between EPSPs, which facilitated their summation. These results explain how pharmacological neuromodulation of the spinal motoneuron networks enhances the electrically evoked motor activity observed in animals and humans (Courtine et al., 2009; Gerasimenko et al., 2015). This also supports our conclusions on the mechanism of generating steadier and stronger motor outputs.

## Discussion

The current study provides comprehensive investigation of the plasticity, integration, and neuromodulation of electrically evoked sensorimotor inputs in spinal motoneurons, and the resultant motor output in absence of supraspinal inputs. Using electrophysiological recordings and computer simulations, we show that integration of electrically evoked sensory and motor inputs, despite having different plasticity patterns, help generate a stronger and steadier motor output, which is more readily achievable at higher neuromodulatory states. Our data revealed, for the first time, contrasting types of summation between the synaptic and system levels. In motoneurons, sensory and motor EPSPs undergo sublinear summation due to reduction in the driving force of the sensory and motor synaptic currents during their concurrent activation. Nonetheless, the amplitude of the generated sensorimotor EPSPs are large enough to maintain the motoneuronal membrane potential above the firing threshold; thereby increasing the number of motoneurons recruited by each stimulus in the train. This leads to two functional outcomes: The coAPs evoked by motoneurons at the ventral roots, compared to those generated by either input separately, become larger and steadier in amplitude throughout the stimulation train. This leads to a stronger and more stable spinal motor output. Accordingly, these results provide, for the first time, mechanistic explanation for the cellular processes contributing to the functional motor improvement observed in subjects with complete SCI when electrical stimulation is delivered. Additionally, these data could potentially guide and/or refine the design of more effective stimulation protocols in patients with SCI, ultimately improving the restoration of motor control and patient independence.

### Short-Term Plasticity of Sensory and Motor Inputs

Repeated activation of synapses can result in either gradual increase or decrease in the resulting synaptic current in the postsynaptic cell. This phenomenon is known as use-dependent (or short-term) plasticity, which is a hallmark of synaptic transmission in the nervous system (Zucker and Regehr, 2002; Fioravante and Regehr, 2011). This form of synaptic plasticity is caused by changes in the Ca^2+^ dynamics and the probabilistic vesicular release at the presynaptic terminal (Neher and Sakaba, 2008; Regehr, 2012). We characterized the short-term changes in synaptic activity of the electrically triggered sensory and motor inputs to spinal motoneurons at physiological frequencies. The excitatory potentials generated in motoneurons by electrical stimulation of the dorsal roots (the sensory input) are dominated by the monosynaptic glutamatergic connections of the Ia afferents (Pinco and Lev-Tov, 1993; Jiang et al., 2015). A train of stimulation to the dorsal roots results in gradual depression of the evoked response. These results agree with published data that showed a similar pattern for sensory inputs *in vitro* (Lev-Tov and Pinco, 1992; Barriere et al., 2008; Jiang et al., 2017). This depression was only partially relieved when we blocked synaptic inhibition, and it persisted when polysynaptic transmission is blocked by mephenesin (Lev-Tov and Pinco, 1992). This indicates a role for reduced transmitter release from vesicles in short-term depression. Reduced vesicular release is commonly seen in synapses with high initial release probability (Alabi and Tsien, 2012), evident by the large-amplitude of the 1st EPSP in a train of stimuli. In agreement with this explanation, the plasticity pattern of this Ia afferent-motoneuron synapse was reversed to facilitation by lowering the extracellular calcium (Pinco and Lev-Tov, 1993). With low Ca^2+^, the 1st EPSP of the train becomes smaller and the subsequent ones are progressively larger, due to build-up of Ca^2+^ in the synaptic terminals.

The motor inputs were activated by stimulation of the local motor circuits and remaining descending axons in the ventral funiculus of the sacral cord. Only a few descending tracts reach the sacral cord, including the LVST (Gossard et al., 1996; Liang et al., 2014). Fibers of the LVST originate in the lateral vestibular nucleus and travel the entire length of the spinal cord, where they synapse mainly onto ipsilateral ventral horn neurons in the mouse, rat, and cat (Gossard et al., 1996; Kuze et al., 1999; Bacskai et al., 2002; Liang et al., 2014). A few other descending tracts originating in the oral pontine reticular nucleus (PnO), and gigantocellular reticular nucleus (Gi) have also been traced down to the sacral cord of the mouse (Liang et al., 2015, 2016). However, the Gi fibers project bilaterally in the spinal cord (Liang et al., 2016) and the PnO sends only a small number of fibers to the lower segments of the spinal cord (Liang et al., 2015). Because we usually observed strong ipsilateral response to descending stimulation, we posit that the response is generated primarily by activation of the LVST fibers in addition to local interneurons.

The synaptic potentials and effective synaptic currents generated by the motor input were generally smaller than those generated by the sensory input. This is in agreement with studies of cat motoneurons which showed that cells with high input resistance have smaller effective synaptic currents from the LVST than from Ia afferents (Binder et al., 2000). The motor input EPSPs had a similar delay to the sensory response, but with longer time-to-peak and half-decay time. This could be the result of fusion of multiple EPSPs, as the response has a pronounced polysynaptic component (Jiang et al., 2015). Alternatively, the synapses might be located more distally on the motoneuron dendrites (Magee, 2000; Spruston, 2008). The slow decay of the inputs increases the chance for integration, which would be more influential when other synaptic inputs have different onset.

Upon successive stimulation of the descending inputs, motor EPSPs showed facilitation, resulting in gradual enhancement of coAPs in the ventral roots. Interestingly, the membrane potential between EPSPs at different pulses was depolarized, and sometimes remained elevated for few seconds after the train. The facilitation of the descending response, thus, could be due to: (1) short-term synaptic facilitation (STF), caused by gradual accumulation of Ca^2+^ in the synaptic terminals, or (2) increased background network excitation resulting from summation of asynchronous EPSPs (Jiang et al., 2015), or a combination of both.

Of note, with either input, the ventral root coAPs show larger changes than the EPSPs recorded in single motoneurons (discussed below). When the two inputs are combined, two features of the resulting sensorimotor response are notable. First, opposite summation styles between the EPSPs (synaptic level) and coAPs (system level) is observed: sublinear summation of EPSPs vs. supralinear summation of coAPs. Second, the motor output of the integrated sensorimotor inputs is larger and steadier (i.e., without adaptation during stimulation) than the individual responses.

### Contrasting Summation Between the Synaptic and System Levels

Sublinear summation of EPSPs has been reported in different neuronal types (Wolf et al., 1998; Magee, 2000). In cat motoneurons, summation of EPSPs is slightly but significantly sublinear (Binder and Powers, 1999; Powers and Binder, 2000). This effect could be due to increased cell conductance upon activation of synaptic ion channels, a decrease in the driving force of the synaptic current, or both. Our measurements of the sensory and motor EPSPs at different holding currents showed no change in the slope of the current/voltage relationship of the cell, indicating no change in the total cell conductance. To test the effect of the driving force, we used a computational model of a single motoneuron to study the integration of synaptic potentials with characteristics incorporated from our experimental data. Two findings are notable from these simulations: (1) Summation of the sensory and motor EPSPs is always sublinear, and the degree of sublinearity is proportional to the driving force, and (2) sublinearity increases when the synaptic conductance is increased (i.e., summation of large-amplitude EPSPs would be more sublinear than that of small-amplitude EPSPs). In fact, sublinearity in our experimental data at 10×T is more pronounced than that at 1.5×T, confirming this trend.

Despite this sublinear summation of EPSP amplitudes at the synaptic level, the increased amplitude of the integrated S&M EPSPs is large enough to maintain the motoneuron membrane potential above the firing threshold. Thus, the probability of the motoneuron firing APs by electrical stimuli becomes much higher than the sum of the probability of each separate input (i.e., supralinear summation). In other words, the motoneuronal firing threshold filters out subthreshold sublinearly summating synaptic events, and only converts probabilistic supra-threshold EPSPs into system events, effectively acting as an amplitude-selective filter separating the synaptic and system levels. This explains the mismatch in summation and plasticity between EPSPs and coAPs observed in the present study. This also explains the effectiveness of including proprioceptive sensory feedback to induce stepping and standing in patients with complete SCI via spinal cord stimulation (Harkema et al., 2011; Angeli et al., 2018): The S&M combination and amplitude-selection-filter effect enhanced the probability of supra-threshold EPSPs generation. This study demonstrates the significant impact the motoneuronal firing threshold has on the transformation of electrically evoked synaptic potentials into motor system output. The simulations showed that minor shifts in the firing threshold (in the order of 0.5 mV) would have drastic effects on the generated motor output.

### Effects of the Neuromodulatory State

In this study, we examined the effect of neuromodulation on short-term synaptic plasticity of the sensory and motor inputs to spinal motoneurons. Methoxamine, the α_1_-adrenergic receptor agonist, has been shown to increase the excitability of the spinal motor networks (Lee and Heckman, 1999; Gabbay and Lev-Tov, 2004; Rank et al., 2011; Mahrous and Elbasiouny, 2018). In our data, methoxamine-induced neuromodulatory state resulted in less sensory depression, more descending facilitation, and even more stable integrated output. This provides the mechanism behind the enhancement of the effect of spinal stimulation when combined with pharmacological neuromodulation (Ichiyama et al., 2008; Courtine et al., 2009; Duru et al., 2015; Gerasimenko et al., 2015).

This study mechanistically highlights the role of sensorimotor integration in generating steady motor outputs. Our data could explain the need to combine spinal cord electrical stimulation with peripheral sensory feedback (generated during motor training) to successfully induce stepping and standing in patients with complete SCI. In addition, our results support that this effect could be boosted by pharmacological neuromodulators. The data also suggest that dorsal root stimulation might be combined with spinal cord stimulation to improve the clinical outcome in patients who failed to independently stand and step with epidural stimulation alone. Taken together, the mechanistic understanding provided by these results is expected to guide the field in designing more refined and effective electrical stimulation interventions, with the ultimate goal of maximizing restored movement, quality of life, and independence in SCI patients.

## Data Availability

All datasets generated for this study are included in the manuscript and/or the Supplementary Files.

## Ethics Statement

This study was carried out in accordance with the recommendations of Wright State University Animal Care and Use Committee. The protocol was approved by the Wright State University Animal Care and Use Committee.

## Author Contributions

AM and SE conceived the presented idea, discussed the results, and verified the analytical methods. AM planned and carried out the experimental work, and drafted the manuscript. MM and SE developed the computer models and performed the computer simulations. AM, MH, and SE edited and approved the final manuscript.

## Conflict of Interest Statement

The authors declare that the research was conducted in the absence of any commercial or financial relationships that could be construed as a potential conflict of interest.
