# Supplementary material for: The Mechanistic Basis for Successful Spinal Cord Stimulation to Generate Steady Motor Outputs
Source: Front Cell Neurosci. 2019 Aug 9;13:359. doi: 10.3389/fncel.2019.00359 (PMC6698793; doi:10.3389/fncel.2019.00359)
Supplement: Supplementary file 1 [file Data_Sheet_1.pdf]

Supplementary Material

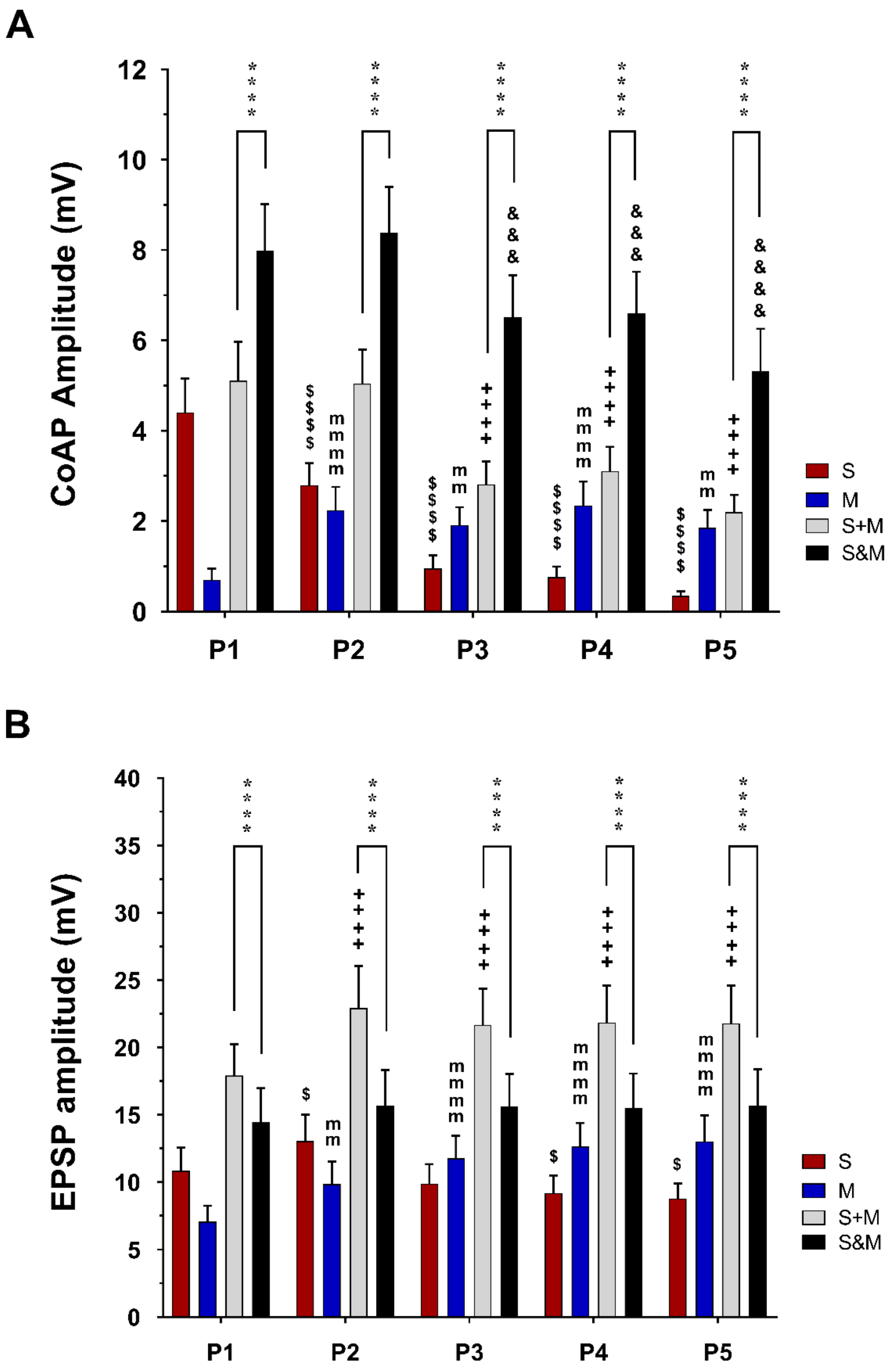

Supplementary Figure 1. Summary of the responses to individual and integrated sensory and motor inputs at 1.5xT/50 Hz stimulation.

Summary of responses in the ventral roots (A) and single motoneurons (B) to 5-pulse electrical stimulation (P1-P5) at a frequency of 50 Hz delivered to the dorsal roots (sensory inputs, '*S'*), descending fibers (motor inputs, '*M*'), or both (sensorimotor integrated inputs, '*S&M*'). The data is presented in the same layout as fig. 4 and fig. 5. **A**: Similar to the response at 25 Hz, coAPs generated by the sensory inputs at 50 Hz exhibit depression while those generated by the motor inputs (M) exhibit facilitation. When compared to the linear summation of the two inputs (S+M), the simultaneous activation of the two inputs (S&M) results in a steadier motor output, and supralinear summation of the coAPs (S&M > S + M), n=15. **B**: At the cellular level, the EPSPs generated by each individual pathway follow the same adaptation pattern as the coAP, though less dramatically. The integration of the two inputs results in non-adapting synaptic potentials, and sublinear summation of the EPSPs (S + M > S&M), n= 9. Data represented as the mean ± SEM. Repeated-measures one-way ANOVA was used to study the pattern of adaptation of each input. Repeated-measures two-way ANOVA was used to test the type of integration (S&M vs. S + M). *The symbol '$' denotes significant difference from S P1, 'm' denotes significant difference from the M P1, '+' denotes significant difference from S + M P1, '&' denotes significant difference from S&M P1, and '*' denotes significant difference between S&M and S+M.*

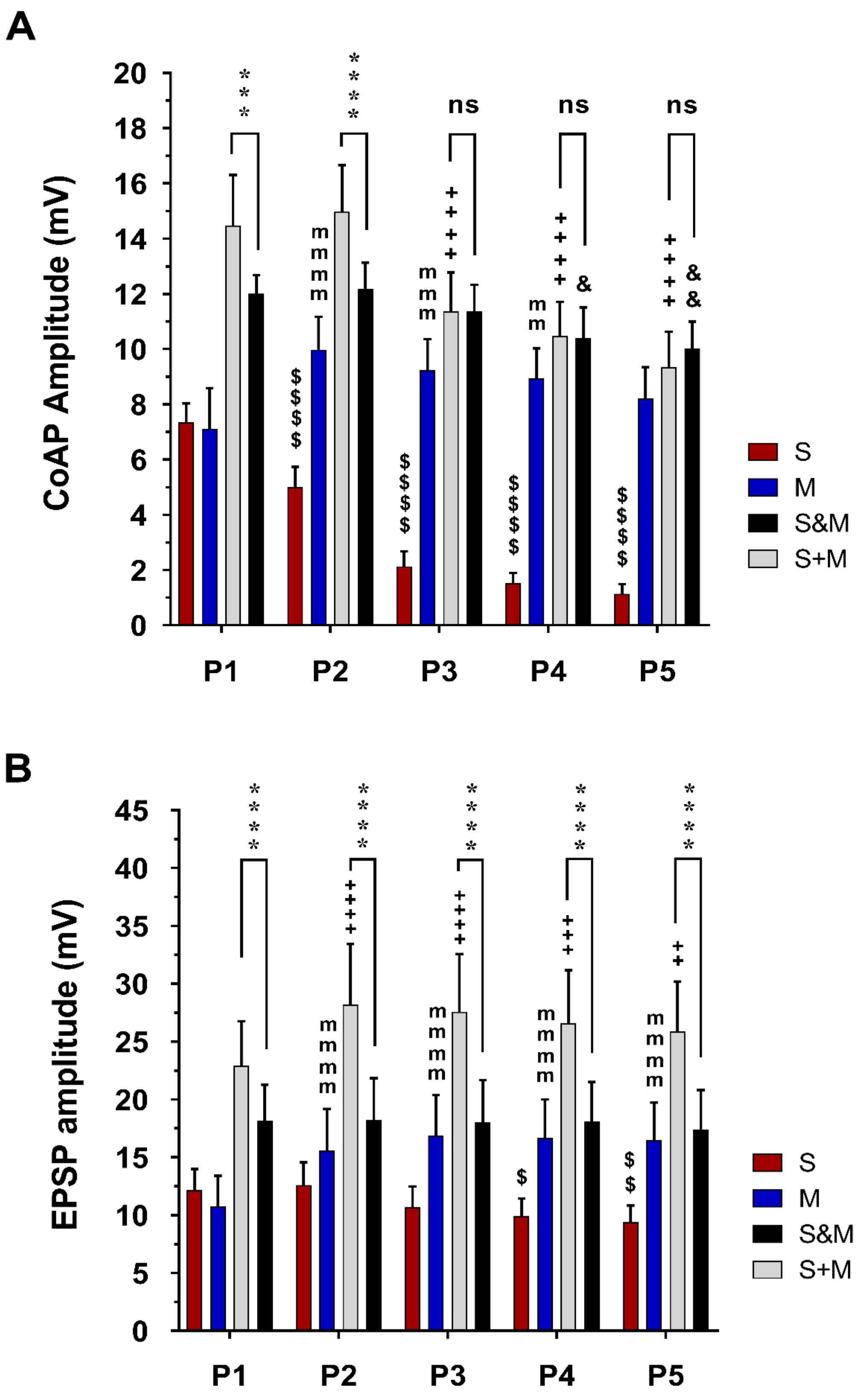
A
CoAP Amplitude (mV)
0
2
4
6
8
10
12
14
16
18
20
P1
P2
P3
P4
P5
ns
ns
ns
S
M
S&M
S+M
B
EPSP amplitude (mV)
0
5
10
15
20
25
30
35
40
45
P1
P2
P3
P4
P5
S
M
S&M
S+M

Supplementary Figure 2. Summary of the responses to individual and integrated sensory and motor inputs at 10xT/50 Hz stimulation.

Summary of responses to 10xT/50 Hz electrical stimulation of the sensory and descending pathways (The layout is similar to fig. 4, fig. 5, and suppl. figure 1). **A**: In the ventral roots, the responses follow the same adaptation patterns as the lower stimulation intensity. The integration of the two inputs results in a steadier motor output and linear summation of the coAPs, except at P1 and P2, where it is sublinear (S + M ≥ S&M), n= 15. **B**: In motoneurons, integration of the two inputs results in non-adapting synaptic potentials (S&M) and sublinear summation (S + M > S&M), n=9. Data representation, statistical analysis, and significance symbols are the same as in fig. 5 and suppl. fig. 1.

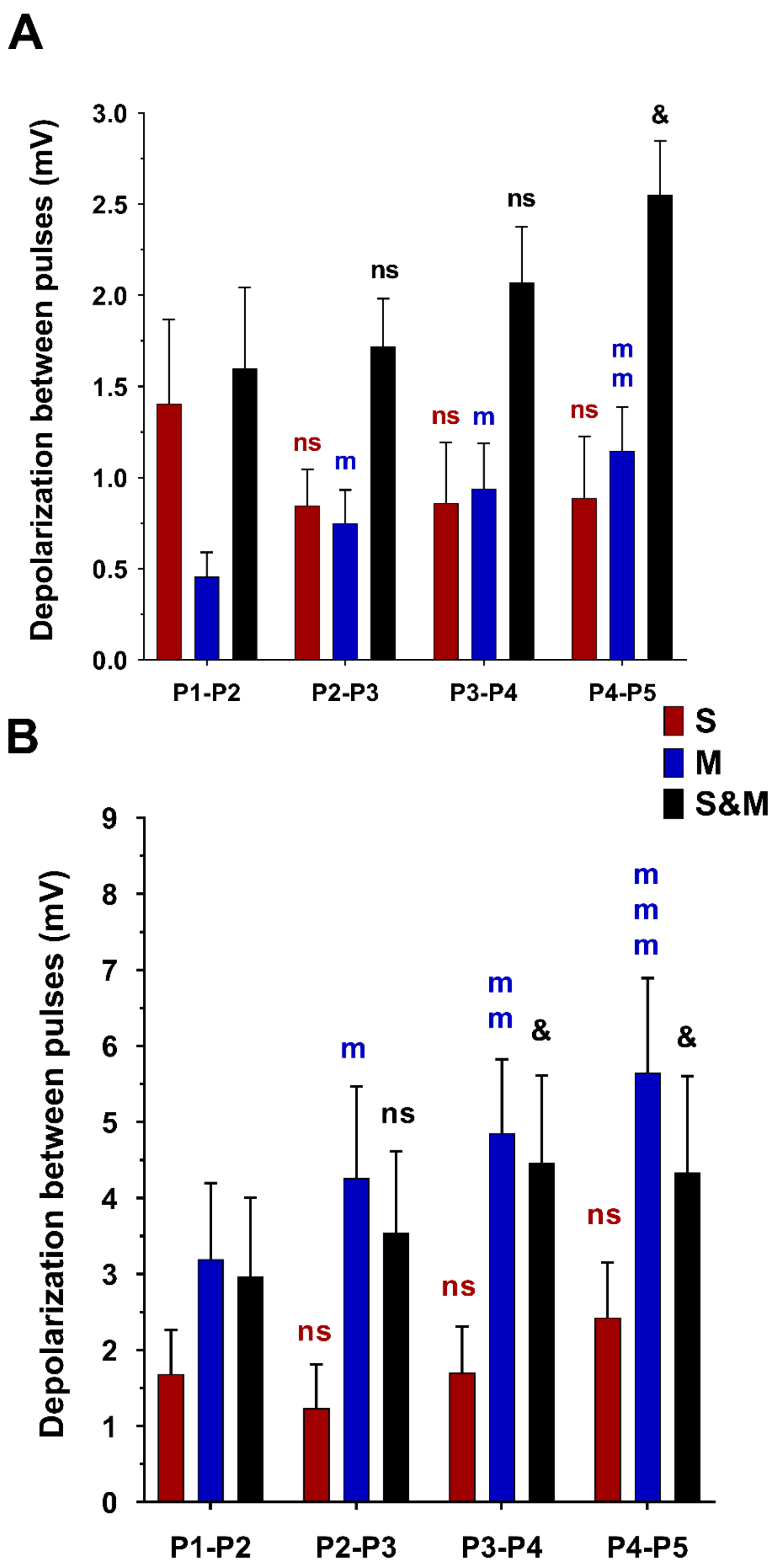
A
Depolarization between pulses (mV)
3.0
2.5
2.0
1.5
1.0
0.5
0.0
&
ns
ns
ns
ns
ns
m
m
m
m
m
P1-P2
P2-P3
P3-P4
P4-P5
B
S
M
S&M
Depolarization between pulses (mV)
9
8
7
6
5
4
3
2
1
0
m
m
m
m
m
m
&
&
ns
ns
ns
ns
P1-P2
P2-P3
P3-P4
P4-P5

Supplementary Figure 3. Changes in the membrane potential of motoneurons in-between pulses of the stimulation train at 25 Hz.

Membrane depolarization between pulses of the stimulation train was measured immediately before the subsequent pulse in respect to the resting potential before the train of stimulation. These changes were quantified at 1.5xT intensity (A) and 10xT intensity (B) for dorsal root stimulation (sensory inputs, red bars labeled '*S*'), descending stimulation (motor inputs, blue bars labeled '*M*'), or both (sensorimotor integrated inputs, black bars labeled '*S&M*'), n=12/each. With the sensory inputs (S), the membrane sustained about 1.5 mV depolarization between P1 and P2; and did not significantly change afterwards. In case of motor stimulation (M), the membrane consistently undergoes progressive depolarization. Similarly, the integrated input (S&M) tended to depolarize progressively which becomes significant with later pulses. This pattern in the integrated input is apparently driven by the motor input. Repeated-measures one-way ANOVA was used to elucidate the changes in membrane potential between the pulses in comparison to the first change between P1 and P2. *'ns' denotes non-significant differences, 'm' denotes significant difference from the M P1-P2, '&' denotes significant difference from S&M P1-P2.*
